# Oxidative Stress-Related Signaling Pathways Predict Oocytes’ Fertilization In Vitro and Embryo Quality

**DOI:** 10.3390/ijms232113442

**Published:** 2022-11-03

**Authors:** Paolo Giovanni Artini, Giorgia Scarfò, Ilaria Marzi, Jonathan Fusi, Maria Elena Obino, Ferdinando Franzoni, Elisa Zappelli, Elisa Chelucci, Claudia Martini, Vito Cela, Simona Daniele

**Affiliations:** 1 Division of Gynecology and Obstetrics, Department of Clinical and Experimental Medicine, University of Pisa, 56100 Pisa, Italy; 2 Division of General Medicine, Department of Clinical and Experimental Medicine, University of Pisa, 56100 Pisa, Italy; 3 Department of Pharmacy, University of Pisa, 56100 Pisa, Italy

**Keywords:** antioxidant capability, oxidative stress, inflammation, oocytes fertilization, embryo quality

## Abstract

Oocyte development and fertilization are largely influenced by the microenvironment of the follicular fluid (FF), and the exploration of its molecular/metabolic composition may help in improving in vitro fertilization (IVF) outcomes. Here, the concentrations of molecules related to oxidative stress/inflammation were measured in FF from follicles at oocyte retrieval during IVF. Here, the FF antioxidant potential was correlated with the number of retrieved/mature oocytes and the number of fertilized ones. FF collected from the follicles of normal fertilized oocytes presented an elevated antioxidant capability, lower levels of pro-inflammatory molecules (i.e., IL-6, IL-8, IL-12, TGF-β, and HIF-1α), and a higher IL-10 concentration. FF samples from follicles at oocyte retrieval that resulted in top-quality embryos displayed a peculiar antioxidant capability and a further decrease in proinflammatory molecules when compared with FF, giving rise to poor-quality embryos. Finally, pro-inflammatory molecules were lower and accompanied by a high antioxidant capability in samples giving rise to successful embryo implantation. The antioxidant capability and IL-10 displayed a good predictive ability for fertilization and embryo quality. Overall, our data showed the great influence of oxidative stress on the oocytes’ fertilization, and shed light on the importance of controlling the inflammatory and oxidative status of FF to obtain good-quality embryos with significant implantation potential.

## 1. Introduction

Despite advances in assisted reproduction treatment (ART), in vitro fertilization (IVF) techniques have a positive outcome in a limited number of cycles [1,2]. The investigation of factors affecting IVF/ICSI outcomes may help to improve the success rates [2]. Among these factors, oocyte quality influences the effectiveness of oocyte fertilization, and affects the embryo development of fertilized oocytes. Thus, only a small number of oocytes retrieved in an IVF cycle develop into a viable embryo suitable for implantation [3].

Recently, precise, non-invasive, and cost-effective predictive tests of oocytes’ or embryos’ developmental potential have been developed to increase pregnancy rates in single-embryo transfers [4]. Moreover, since oocyte development is largely influenced by the microenvironment of the follicular fluid (FF) [5], recent studies have explored the molecular and metabolic composition of FF potentially affecting oocyte fertilization and embryo quality [6], with undoubted advantages related to the fact that FF can be easily achieved during oocyte retrieval. Hence, FF represents an optimal biological fluid to provide novel candidates that can be used to assess oocytes’ competence and embryos’ implantation ability [7,8,9,10].

Among the metabolic products of the granulosa and theca cells contained in FF, particular interest has been focused on oxidation stress, which has been proposed as one of the most important factors adversely impacting ART outcomes [8]. An imbalance between ROS and the antioxidant defense system in the FF can trigger damage to the DNA, cytoskeleton, and cell membrane, and thus to anomalous oocyte development, finally giving rise to a low oocyte quality and fertilization potential [10]. FF can reflect the metabolic and hormonal processes occurring in the microenvironment of maturing oocytes, and thus it contains biochemical components including cytokines, chemokines, growth factors, and steroid hormones [11]. In this sense, transcriptomic analyses of human FF have shed light on the omics changes associated with oocyte development; nevertheless, the molecular and metabolic signatures of embryogenesis and the related key predictors in FF remain to be investigated [12,13]. Furthermore, conflicting results have been reported on the role of oxidative stress and inflammation in embryogenesis and embryo implantation [14]. On this basis, the objective of the present study was to measure the levels of ILs and the molecules related to oxidative stress in the FF of oocytes retrieved during IVF cycles, and to determine their association with fertilization rate, embryo quality, and embryo implantation. Determination of the metabolites in FF may be useful to understand the high rate of IVF failures and to develop effective pharmacological treatments.

## 2. Results

### 2.1. Baseline Clinical Characteristics of Patients

In total, 107 FF samples were collected from similarly mature oocytes retrieved from women undergoing ART cycles and were included in the present study, as reported in the Methods section. The samples were further divided into FF from oocytes with normal fertilization (F) and FF from oocytes with abnormal fertilization or that failed to fertilize (NF). Among the FF from oocytes with normal fertilization, samples were further divided according to the quality status of Day 3 embryos as FF from top-quality embryos (TQ) and FF from impaired or poor-quality embryos (NTQ). Of the 107 FF samples, 92 (85.9%) had successful fertilization. Of the fertilized oocytes, 55 (59.8%) developed into TQ embryos (51.4% of all oocytes).

The group of 52 patients undergoing IVF treatment was homogenous, as shown in Table 1. We found that age (*p* = 0.4230; 0.9756); BMI (*p* = 0.3957; 0.8208); and levels of AMH (*p* = 0.5131; 0.3660), FSH (*p* = 0.9824; 0.2017), LH (*p* = 0.6024; 0.8308), and E2 (*p* = 0.0692; 0.9235) were comparable between the subgroups of patients. AFC (*p* = 0.1614; 0.1610), total units of gonadotropins (*p* = 0.3508; 0.6224), days of stimulation (*p* = 0.6943; 0.6947), progesterone (*p* = 0.6792; 0.4298), and estradiol (*p* = 0.5504; 0.1564) on the trigger day were also comparable between the subgroups.

Spearman’s correlation revealed the positive association of age and BMI with the total units of gonadotropins (age: *p* = 0.0039; BMI: *p* < 0.001) and days of stimulation (age: *p* = 0.3404; BMI: *p* < 0.001). As expected, AMH and AFC were negatively but significantly related to the total units of gonadotropins (AMH: *p* = 0.0001; AFC: *p* = 0.0101) and days of stimulation (AMH: *p* = 0.0118; AFC: *p* < 0.0001), and positively related with the number of retrieved oocytes (AMH: *p* < 0.0001; AFC: *p* = 0.0008) and mature oocytes (AMH: *p* < 0.0001; AFC: *p* = 0.0038).

Interestingly, BMI was also significantly and negatively associated with the number of follicles > 16 mm (trigger day) (BMI: *p* = 0.0201), retrieved oocytes (BMI: *p* = 0.0522), and mature oocytes (BMI: *p* = 0.0148). In contrast, estradiol value on the trigger day positively influenced the number of retrieved oocytes (estradiol on the trigger day: *p* < 0.0001) and mature oocytes (estradiol on the trigger day: *p* = 0.0003).

Regarding the fertilization and embryo quality parameters, the number of fertilized oocytes and the number of top-quality embryos were found to be related positively to AMH (fertilized oocytes: *p* = 0.0026; top-quality embryos: *p* = 0.0409) and estradiol values on the trigger day (fertilized oocytes: *p* = 0.0001; top-quality embryos: *p* < 0.0001). Interestingly, BMI and FSH were inversely correlated with the number of fertilized eggs (BMI: *p* = 0.0082; FSH: *p* = 0.0099).

### 2.2. Concentrations of Biochemical Parameters Related to Inflammation and Oxidative Stress in Follicular Fluid

**Figure 1:**
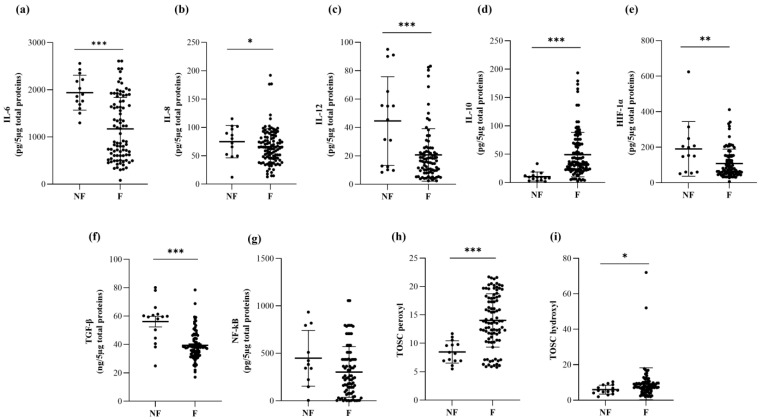
Protein levels of the proinflammatory cytokines IL-6 (**a**), IL-8 (**b**), and IL-12 (**c**); the anti-inflammatory cytokine IL-10 (**d**); the pro-inflammatory factors HIF-1α (**e**), TGF-β (**f**), and NF-kB (**g**); and TOSC values against peroxyl (**h**) and hydroxyl (**i**) radicals in follicular fluid (FF) samples from unfertilized (NF) and fertilized oocytes (F). The interleukins and pro-inflammatory factors were measured with commercial ELISA kits, while the antioxidant activity of FF samples was measured by chromatographic analysis. The data are reported as the mean values ± SD. Statistical analysis was performed by unpaired *t*-tests: * *p* < 0.05, ** *p* < 0.01, *** *p* < 0.001 (TQ vs. NTQ).

The amount of selected inflammatory and anti-inflammatory ILs and the related cytokines was measured in FF derived from unfertilized and fertilized oocytes (Table 2). In our study, the FF derived from fertilized oocytes presented a significantly lower concentration of the proinflammatory cytokines IL-6 (*p* = 0.0003, Figure 1a, Table 2), IL-8 (*p* = 0.0249, Figure 1b, Table 2), and IL-12 (*p* < 0.0001, Figure 1c, Table 2) with respect to unfertilized ones. Consistently, HIF-1α and TGF-β showed a lower concentration in FF derived from fertilized oocytes (HIF-1α: *p* = 0.0013, Figure 1e; TGF-β: *p* < 0.0001, Figure 1f, Table 2).

In contrast, the levels of the anti-inflammatory cytokine IL-10 (*p* = 0.0005, Figure 1d, Table 2) was significantly elevated in FF derived from fertilized oocytes as compared with unfertilized ones. The concentrations of IL-6 were related positively with the levels of the other proinflammatory molecules IL-8 (*p* < 0.0001), IL-12 (*p* < 0.0001), HIF-1α (*p* = 0.0127), and TGF-β (*p* < 0.0001) and inversely related to IL-10 (*p* < 0.0001) content.

The antioxidant activity of FF samples was measured by chromatographic analysis. FF from fertilized oocytes displayed a significantly higher antioxidant capability with respect to that from unfertilized ones (TOSC vs. peroxyl radicals: *p* < 0.0001, Figure 1h; TOSC vs. hydroxyl radicals: *p* = 0.0334, Figure 1i, Table 2), thus indicating that the minor concentration of proinflammatory molecules was accompanied by an elevated antioxidant capability in samples derived from fertilized oocytes.

The TOSC values in the FF were inversely related to the concentrations of the inflammatory molecules IL-6 (TOSC vs. peroxyl radicals: *p* = 0.0002; TOSC vs. hydroxyl radicals: *p* = 0.0026), IL-8 (TOSC vs. peroxyl radicals: *p* = 0.0150; TOSC vs. hydroxyl radicals: *p* = 0.0287), IL-12 (TOSC vs. peroxyl radicals: *p* = 0.0724; TOSC vs. hydroxyl radicals: *p* = 0.0161), TGF-β (TOSC vs. peroxyl radicals: *p* = 0.0009; TOSC vs. hydroxyl radicals: *p* = 0.0006), HIF-1α (TOSC vs. peroxyl radicals: *p* = 0.0069; TOSC vs. hydroxyl radicals: *p* = 0.0025), and NF-kB (TOSC vs. peroxyl radicals: *p* = 0.0431; TOSC vs. hydroxyl radicals: *p* = 0.0423), and proportionally related to IL-10 levels (TOSC vs. peroxyl radicals: *p* < 0.0001; TOSC vs. hydroxyl radicals: *p* < 0.0001).

**Figure 2:**
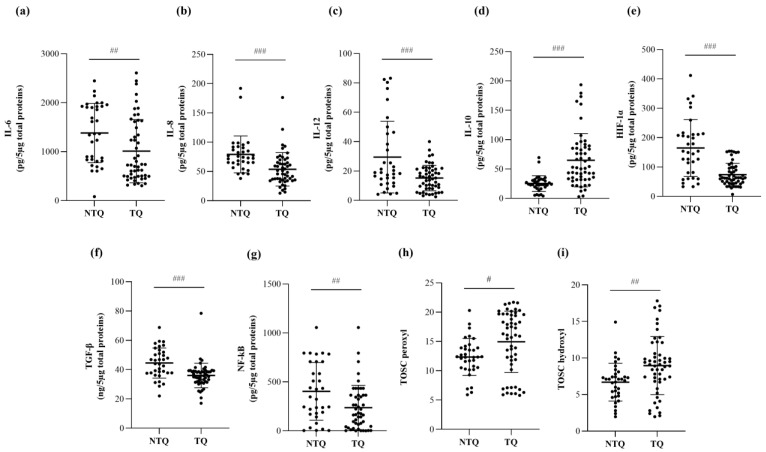
Protein levels of the proinflammatory cytokines IL-6 (**a**), IL-8 (**b**), and IL-12 (**c**); the anti-inflammatory cytokine IL-10 (**d**); the pro-inflammatory factors HIF-1α (**e**), TGF-β (**f**), and NF-kB (**g**); and TOSC values against peroxyl (**h**) and hydroxyl (**i**) radicals in follicular fluid (FF) samples from poor-quality (NTQ) and top-quality (TQ) embryos. The interleukins and pro-inflammatory factors were measured with commercial ELISA kits, while the antioxidant activity (TOSC vs. peroxyl and hydroxyl radicals) of FF samples was measured by chromatographic analysis. The data are reported as the mean values ± SD. Statistical analysis was performed by unpaired *t*-tests: ^#^ *p* < 0.05, ^##^ *p* < 0.01, ^###^ *p* < 0.001 (TQ vs. NTQ).

Among the fertilized oocytes, the statistical analyses were repeated considering the quality of the generated embryos on Day 3. Interestingly, top-quality embryos exhibited significantly lower levels of the proinflammatory ILs IL-6 (*p* = 0.0097, Figure 2a, Table 2), IL-8 (*p* = 0.0002, Figure 2b, Table 2), and IL-12 (*p* = 0.0002, Figure 2c, Table 2), as well as the pro-inflammatory factors HIF-1α (*p* < 0.0001, Figure 2e, Table 2), TGF-β (*p* < 0.0001, Figure 2f, Table 2), and NF-kB (*p* = 0.0055, Figure 2g, Table 2) with respect to poor-quality embryos. In parallel, the TOSC values against hydroxyl radicals (TOSC vs. hydroxyl radicals: *p* = 0.0029, Figure 2i, Table 2) were also elevated in the top-quality embryos.

Finally, the IVF outcome was considered to unveil putative differences in the FF of the fertilized oocytes giving rise to positive or negative β-HCG. Of note, the FF related to positive β-HCG presented a significantly lower content of IL-6 (*p* = 0.0005), IL-8 (*p* = 0.0214), HIF-1α (*p* = 0.0065), TGF-β (*p* = 0.0037), and NF-kB (*p* = 0.0135). In contrast, a higher concentration of IL-10 (*p* < 0.0001) was found. Moreover, TOSC values against peroxyl and hydroxyl radicals were significantly elevated in positive β-HCG samples (TOSC vs. peroxyl radicals: *p* < 0.0001; TOSC vs. hydroxyl radicals: *p* = 0.0003).

Spearman’s correlation revealed that the TOSC values against peroxyl radicals were directly correlated with the number of retrieved oocytes (TOSC vs. peroxyl radicals: *p* = 0.0004), the number of mature oocytes (TOSC vs. peroxyl radicals: *p* < 0.0001), and the number of fertilized ones (TOSC vs. peroxyl radicals: *p* < 0.0001).

Of note, the number of top-quality embryos was negatively influenced by the FF concentrations of IL-6 (*p* = 0.0005), IL-8 (*p* = 0.0136), and IL-12 (*p* = 0.0043), and positively related to IL-10 levels (*p* < 0.0001). Consistently, TOSC values against both peroxyl and hydroxyl radicals were directly in correlation with the number of top-quality embryos (TOSC vs. peroxyl radicals: *p* < 0.0001; TOSC hydroxyl radicals: *p* = 0.0097).

Interestingly, when the subgroup of top-quality embryos was selected, the number of follicles on the trigger day were inversely related to the concentrations of IL-6 (*p* = 0.0174) and TGF-β (*p* = 0.0143), and positively related to those of IL-10 (*p* = 0.0061). Moreover, IL-6 negatively influenced the number of fertilized oocytes (*p* = 0.0248).

### 2.3. Predictive Values of the Biochemical Parameters Measured in the Follicular Fluid for Fertilized vs. Unfertilized Oocytes, and Top-Quality vs. Poor-Quality Embryos

**Figure 3:**
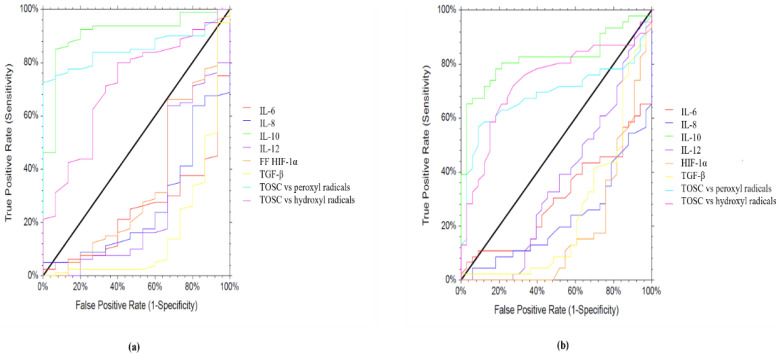
ROC analyses of FF biochemicals’ parameters were carried out to evaluate the capacity to differentiate fertilized oocytes from unfertilized ones (**a**), and top-quality embryos from poor-quality ones (**b**). AUROC and CI values are reported in Table 3.

Logistic regression analysis identified the following variables measured in the follicular fluid as being associated with oocyte fertilization and top-quality embryos: IL-10, TOSC vs. peroxyl radicals, and TOSC vs. hydroxyl radicals (Figure 3).

Regarding oocyte fertilization, for IL-10, the area under the receiving operating characteristic (ROC) curve was 0.91 (cut-off point 95% CL: 0.792; 0.962; *p* < 0.001) with a sensitivity of 87% and a specificity of 74%, while for TOSC vs. peroxyl radicals, the area under the ROC curve was 0.853 (cut-off point 95% CL: 0.759; 0.912; *p* > 0.0001) with a sensitivity: of 81% and a specificity of 70%, and for TOSC vs. hydroxyl radicals, the area under the ROC curve was 0.714 (cut-off point 95% CL: 0.547; 0.826; *p* > 0.001) with a sensitivity of 72% and a specificity of 64%, as shown in Table 3. Regarding embryo quality, for IL-10 the area under the ROC curve was 0.824 (cut-off point 95% CL: 0.702; 0.898; *p* > 0.0001) with a sensitivity of 85% and a specificity of 70%, while for TOSC vs. peroxyl radicals, the area under the ROC curve was 0.687 (cut-off point 95% CL: 0.546; 0.790; *p* > 0.001) with a sensitivity of 72% and a specificity of 61%, and for TOSC vs. hydroxyl radicals, the area under the ROC curve was 0.733 (cut-off point 95% CL: 0.597; 0.828; *p* > 0.0001) with a sensitivity of 78% and a specificity of 67% (Table 3).

## 3. Discussion

In the present study, women undergoing IVF cycles were monitored for their clinical parameters; the concentrations of molecules related to oxidative stress and inflammation were measured in the follicular fluids (FF) of oocytes retrieved during IVF cycles, with the objective of determining their association with the fertilization rate, embryo quality, and embryo implantation. The main findings are as follows. First, the number of fertilized oocytes and the number of top-quality embryos were found to be related positively to AMH and estradiol values on the trigger day, and BMI and FSH were inversely correlated with the number of fertilized eggs. Second, the antioxidant potential of FF was directly correlated with the number of retrieved and mature oocytes, and the number of fertilized ones. Third, FF from oocytes with normal fertilization presented an elevated antioxidant capability, lower levels of pro-inflammatory molecules (i.e., IL-6, IL-8, IL-12, TGF-β, and HIF-1α), and a higher concentration of IL-10. Fourth, the number of top-quality embryos was negatively influenced by the FF concentrations of IL-6, IL-8, and IL-12, and positively related to IL-10 levels. Fifth, among the FF samples from oocytes with normal fertilization, FF samples from top-quality embryos displayed a peculiar antioxidant capability and a further decrease in proinflammatory molecules when compared with FF giving rise to poor-quality embryos. Lastly, samples giving rise to successful embryo implantation showed significantly lower concentrations of pro-inflammatory molecules and a higher antioxidant capability compared with unsuccessful implantation.

Overall, our data showed the great influence of oxidative stress on the oocytes’ fertilization, and shed light on the importance of controlling the inflammatory and oxidative status of FF to obtain good-quality embryos with significant implantation potential.

Follicular fluid retains several molecules, including sugars, ROS, and hormones, which directly affect the developmental competence, maturation ability, and the quality of oocytes [14,15]. In particular, an imbalance between antioxidant molecules and ROS production in FF during ovulation has been suggested to influence oocyte quality, fertilization, and embryo development [16]. Considering that ovarian stimulation itself can induce an inflammatory response, the determination of metabolites in FF may be useful to understand the high rate of IVF failures and to develop potentially effective pharmacological treatments.

In the present study, 55 women were enrolled and monitored for clinical and biochemical parameters during IVF/ICSI cycles. Serum estradiol value on the trigger day positively influenced the number of retrieved oocytes and mature oocytes, as well as the number of fertilized oocytes and the number of top-quality embryos, consistent with previous reports [17,18].

FF from fertilized oocytes displayed a significantly higher antioxidant capability compared with unfertilized ones, thus indicating that the minor concentration of proinflammatory molecules is accompanied by elevated antioxidant capability in samples derived from fertilized oocytes. Consistently, the total antioxidant capacity of FF has been shown to be significantly higher in follicles bearing oocytes that are competent for fertilization than in follicles with non-fertilizable oocytes [19]. Overall, the impact of oxidative stress on oocyte maturation seems to be deleterious, although conflicting results exist. In this sense, surprisingly, a few studies have found a positive correlation between FF ROS levels and maturation parameters [20,21].

In our case, FF derived from fertilized oocytes presented a significantly lower concentration of the proinflammatory cytokines (IL-6, IL-8, and IL-12) and the related inflammatory molecules (TGF-β and HIF-1α) with respect to the unfertilized oocytes. Moreover, the level of the anti-inflammatory cytokine IL-10 was significantly elevated in FF derived from fertilized oocytes compared with unfertilized ones. The concentrations of IL-6 were correlated positively with the levels of the other proinflammatory molecules (IL-8, IL-12, HIF-1α, and TGF-β) and inversely related to IL-10 content. Overall, our data suggest that a low-proinflammatory microenvironment facilitates oocytes’ fertilization. In accordance with our data, failed fertilization has been reported in women with detectable IL-12 [22]. In contrast, high levels of intrafollicular IL-1β have been associated with good fertilization rates [23], while no correlation has been reported between the preovulatory FF concentration of IL-10 or IL-8, and both oocyte numbers and the fertilization rate [22,24]. Overall, the data in the literature report conflicting results on the association between inflammatory molecules and fertilization rates. Such differences may be related to the processing of FF and data expression. Moreover, another parameter impacting the results may be represented by the median age of women enrolled in the different studies. Finally, besides the fertilization method, additional bias related to the type of infertility may be present.

In the fertilized oocytes, TOSC values against both hydroxyl and peroxyl radicals were directly correlated with the number of top-quality embryos. Consistently, total antioxidant capability has been shown to have a positive correlation with embryo quality and with pregnancy rates in IVF [25]. The presence of higher antioxidant amounts in FF than in the serum can reflect the antioxidant activity of granulosa cells [26], thus constituting a marker of mature follicles leading to the growth of high-quality oocytes [14].

Moreover, here, top-quality embryos exhibited significantly lower levels of the proinflammatory ILs (IL-6, IL-8, and IL-12), as well as of the pro-inflammatory factors TGF-β, HIF-1α, and NF-kB compared with poor-quality embryos. Consistently, FF IL-1β levels have been reported to be lower in FFs for which the oocytes were able to generate better embryos and successful IVF attempts [27], and elevated levels of IL-2 and interferon (IFN-γ) have been revealed in the FF of follicles corresponding to highly fragmented embryos and early-cleaving embryos [28]. In contrast, other studies have reported no correlation between IL-1β and IL-10 concentrations and embryo quality [24].

Logistic regression analysis revealed that the antioxidant activity of follicular fluid is a good predictor of oocyte fertilization and top-quality embryos. Both TOSC vs. peroxyl radicals and TOSC vs. hydroxyl radicals demonstrated high sensitivity and specificity, accordingly to previous published data [29]. Similarly, IL-10 displayed a good predictive ability for fertilization and embryo quality, thus revealing the importance of maintaining an oocyte microenvironment with a specific anti-inflammatory status.

Finally, IVF outcome was considered to unveil putative differences in the FF of the fertilized oocytes giving rise to positive or negative β-HCG. TOSC values against hydroxyl and peroxyl radicals were significantly elevated in positive β-HCG samples. Previous reports have shown that the total antioxidant capacity of FF or serum is positively correlated with the pregnancy rate [30]; Of note, in our case, FF related to positive β-HCG presented a significantly lower content of IL-6, IL-8, HIF-1α, TGF-β, and NF-kB. In contrast, this FF had a higher concentration of IL-10. Consistently, the IL-12 in FF appears to be associated with a negative outcome of IVF treatment [22]. In contrast, other studies have reported no correlation between IL-1β and IL-10 concentrations and the pregnancy rate [22,24]. Surprisingly, a recent study has even reported that the concentration of IL-6 in the follicular fluid is elevated in patients with confirmed pregnancy [31], thus highlighting the heterogeneity of the results in the literature. In this sense, the different stimulation protocols used during controlled ovarian stimulation may also play a role, since it has been established that gonadotrophin stimulation in IVF alters the immune cell profile in the follicular fluid and the cytokine concentrations in the follicular fluid and serum [32].

## 4. Materials and Methods

### 4.1. Patients

Patients undergoing IVF treatment from June 2019 to December 2020 were enrolled at the Center of Infertility and Assisted Reproduction of the Department of Clinical and Experimental Medicine of Pisa. All patients older than 37 years of age were excluded; other exclusion criteria were: smoking habit, karyotype abnormalities, or a history of endometriosis, ovarian hyperstimulation syndrome (OHSS), repeated embryo implantation failures, ectopic pregnancy, ovarian surgery, or benign or malignant ovarian tumors. We collected 55 patients who had subjected to IVF/ICSI and embryo culture, although 3 of them did not perform the embryo transfer. As a consequence, 52 cases completed the procedure, and their pregnancy outcomes were monitored. The study was conducted according to the guidelines of the Declaration of Helsinki and approved by the Ethics Committee (CTO, Clinical Trial Office) of Azienda Ospedaliero Universitaria Pisana (AOUP) (protocol code 35,105, approved on 13 June 2019) [33,34].

After a complete clinical history and measurement of the anthropometric parameters including age, height, weight, and body mass index (BMI), biochemical analyses and transvaginal ultrasonography (US) were performed. In addition, patients underwent fertility investigations which consisted of hysterosalpingography; hysteroscopy; Cycle Day 3 measurements of the serum levels of estradiol (E2), follicle-stimulating hormone (FSH), and anti-Müllerian hormone (AMH); transvaginal US with antral follicle count (AFC); and semen analysis for the partner. Patients who showed endocrine dysfunctions such as polycystic ovary syndrome, hyperprolactinemia, thyroid dysfunction, hypothalamic amenorrhea, Cushing’s syndrome, and congenital adrenal hyperplasia were excluded as well. The women has been infertile for at least 1 year.

### 4.2. IVF Procedures

Institutional clinical protocols were applied in order to monitor and manage the IVF procedures. Patients were administered 150–450 IU/day of recombinant FSH or hMG (human menopausal gonadotropin) using a gonadotropin-releasing hormone (GnRH) antagonist protocol (or long agonist protocol) to achieve controlled ovarian hyperstimulation (COH). The COH scheme, as well as the initial dose of gonadotropin, were determined by taking women’s clinical features into account including age, BMI, AFC, and AMH levels. In order to prevent an early increase in LH, when the lead follicle measured 12–14 mm, patients were given 0.125 mg/d of Cetrorelix (Cetrotide, Merck Serono Spa, Rome, Italy), a GnRH antagonist.

When the diameter of 1–2 follicles measured 17 mm, the maturity of the final oocyte was obtained by administering a dose of 250 mg of recombinant HCG (Ovitrelle, Merck Serono Europe Ltd., London, UK). After 36 h, patients underwent a transvaginal follicular aspiration to collect the oocytes. The World Health Organization criteria [35] were used to determine the volume, sperm count, forward motility, and morphology. Retrieved oocytes were preserved in an oocyte culture medium (Sydney IVF Oocyte Wash Buffer; Cook Ireland Ltd., Limerick, Ireland). Besides the sperm parameters and the clinical history of patients, ICSI was selected for all couples enrolled here to ensure the utilization of better-quality sperm. In this way, the variability produced by conventional fertilization was nullified. The presence of 2 pronuclei after the procedure confirmed the occurrence of fertilization. Fertilized oocytes were cultured until embryo transfer into a fresh cleavage medium (Sydney IVF Cleavage Medium; Cook Ireland Ltd.). The same embryologist analyzed the embryos on Day 3 (46–48 h after the fertilization) and graded them as I–IV (best to worst). ET was then carried out (Day 3 or 5), under the guidance of abdominal US, using an aK-Soft 500 Embryo Transfer Catheter (Cook, Ireland Ltd.). After ET, to start the luteal phase, patients were given 200 mg of vaginal micronized progesterone three times a day (Prometrium, Rottapharm S.p.A., Milan, Italy) and intramuscular hydroxyprogesterone caproate every 72 h (Lentogest^®^, IBSA Farmaceutici Srl, Lodi, Italy).

### 4.3. Collection of the Follicular Fluid Samples

**Figure 4:**
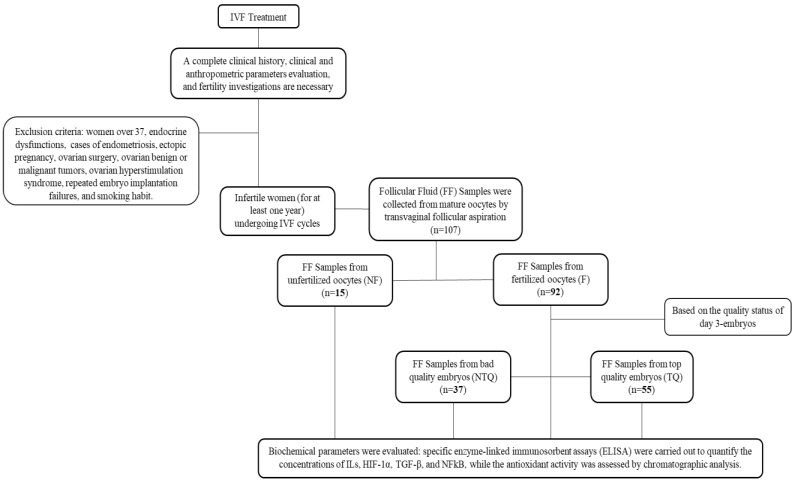
Flow chart of sample collection and analysis of follicular fluid (FF).

Through use of fine needle aspiration on the day of oocyte retrieval, the follicular fluid from each follicle was taken from 55 patients and then centrifuged at 4 °C for 10 min at 2500 rpm (to clear it from cellular components) and preserved at −80 °C until assayed. The samples were further divided into FF from oocytes with normal fertilization (F) and FF from oocytes with abnormal fertilization or that failed to fertilize (NF). Among the FF from oocytes with normal fertilization, samples were further divided by considering the quality status of Day 3 embryos, namely as FF from top-quality embryos (TQ) and FF from impaired or poor-quality embryos (NTQ) (Figure 4).

### 4.4. Measurement of NF-kB in Follicular Fluid

An enzyme-linked immunosorbent assay (ELISA) was used to determine the follicular fluid concentrations of p65 NF-kB, as previously described [33,36]. In brief, plates were pretreated with antibodies against p65 NF-kB diluted in poly-L-ornithine and kept overnight at 4 °C. In order to inactivate nonspecific sites, the plates were treated with 1% BSA for 2 h at 37 °C, after washing with 0.01% PBS-Tween. After adding the follicular fluid and serum for 2 h at 25 °C, the plates were incubated (2 h at 25 °C with polyclonal antibodies against p65 NF-kB (sc-372, Santa Cruz Biotechnology, anti-rabbit) or N-cadherin (sc-7939, Santa Cruz Biotechnology, anti-rabbit). This process was followed by the addition of a specific HRP antibody (Santa Cruz Biotechnology) for 1 h at 37 °C, 3,3′,5,5′-tetramethylbenzidine (TMB), and a stop solution (H_2_SO_4_). Successively, the absorbance was read at 450 nm (EnSight Multimode Plate Reader, PerkinElmer). All measurements were repeated 3 times with specific calibration for each protein.

### 4.5. Measurement of Interleukins (ILs) and TGF-β in Follicular Fluid

Specific enzyme-linked immunosorbent assay (ELISA) kits (Cloud-Clone Corp., Katy, TX, USA: SEA124Hu-96 for TGF-β, SEA079Hu for IL-6, SEA080Hu for IL-8, SEA111Hu for IL-12, and SEA056Hu for IL-10) were used to perform the measurements of TGF-β, and other interleukins in according to the kits’ instructions. Succinctly, suitable wells were incubated with follicular fluid or serum (diluted 1:10 in a standard diluent) for 1 h at 37 °C, then 100 µL of the primary and secondary antibodies was added for 1 h at 37 °C and for 30 min at 37 °C, respectively. Successively, in each well a substrate solution was inserted, allowing the color to evolve for 10–20 min at 37 °C. The absorbance was read at 450 nm [36].

### 4.6. Measurement of HIF-1α in Follicular Fluid

An enzyme-linked immunosorbent assay (ELISA) kit (RAB1057-1KT, Sigma Aldrich, Milan, Italy) was used according to the specific instructions. For this, 100 µL of each standard was incubated with the samples for 2.5 h at room temperature by delicately shaking them. After washing the samples, 100 µL of biotinylated detection antibody were added to the wells for 1 h. Subsequently, 100 µL of a HRP-streptavidin solution was added for 45 min, and the TMB reagent was added for 30 min. Absorbance was measured at 450 nm.

### 4.7. Evaluation of the Total Antioxidant Capability (AOC) in FF

The antioxidant activity of FF samples (NF, F, NTQ, and TQ) was assessed by performing a total oxyradical scavenging capacity (TOSC) assay, a gas chromatographic method usually used in biological samples to evaluate the oxyradical scavenging capacity [34,35,36,37].

Briefly, hydroxyl radicals were obtained by the iron plus ascorbate-driven Fenton reaction (1.8 μM Fe3+, 3.6 μM EDTA, and 180 μM ascorbic acid in 100 mM PBS; pH 7.4) at 35 °C, while the peroxyl radicals were generated by thermal homolysis of 20 mM ABAP in 100 mM PBS (pH 7.4) at 35 °C. Peroxynitrite was produced at 35 °C from the decomposition of SIN-1 in the presence of 0.2 mM KMBA, 100 mM PBS (pH 7.4), and 0.1 mM DTPA at 35 °C. Reactions with 0.2 mM KMBA were performed in 10 mL vials sealed with gas-tight Mininert valves (Supelco, Bellefonte, PA, USA) in a final volume of 1 mL [37,38,39,40,41].

A gas chromatographic analysis of 200 µL aliquots taken from the headspace of vials at timed intervals during the reaction (Hewlett-Packard gas chromatograph, HP 7820A Series, Andoven, M, equipped with a Supelco DB-1 capillary column and a flame ionization detector, FID) was carried out to quantify the ethylene produced from the area under the kinetic curves that best described the experimental points observed for the control reactions and after the addition of the FF samples during the reaction [37,38,39,40,41].

TOSC values were determined via the following equation: TOSC = 100 − (SA/CA × 100), where SA and CA represent the area under the curve (AUC) for the sample and the control reaction, respectively. A TOSC value of 100 indicates the suppression of ethylene formation, while a TOSC value of 0 is linked to the absence of scavenging activity [37,38,39,40,41]. Each experiment was executed in duplicate to allow for the intrinsic variability of the method. The results were expressed in TOSC units.

### 4.8. Statistical Analysis

Data analysis and graphical presentations were performed by using GraphPad Prism software (GraphPad Software Inc., San Diego, CA, USA). Data are expressed as means ± SD. All data were assessed for the normality of distribution using the Shapiro–Wilk test. The differences in the means between oocytes with normal fertilization (F) and oocytes with abnormal fertilization or that failed to fertilize (NF), as well as between poor-quality (NTQ) and top-quality (TQ) embryos for the analyzed biomarkers were estimated using a classical Student’s *t*-test for independent samples. Statistical significance was determined at *p* < 0.05. Correlations between parameters were analyzed using Spearman’s correlation. To evaluate the possible impact of the selected biomarkers on the oocytes’ fertilization and embryo quality, a logistic regression was applied. To determine the analyzed parameters in the follicular fluid that could discriminate between normal and abnormal fertilization as well as good-quality and poor-quality embryos events (the best relationship between sensitivity and specificity), the receiver operating characteristic (ROC) curve was applied. The ROC curves were constructed by plotting the specificity (false positive rate) on the *x*-axis and the sensitivity (true positive rate) on the *y*-axis. The area under the curve (AUC) was also calculated, which measures the accuracy, i.e., the ability of the follicular fluid’s parameters to discriminate between normal and abnormal fertilization as well as between good-quality and poor-quality embryos. For the statistical procedures, StatView (Abacus Concepts, Inc., SAS Institute, Cary, NC, USA) was used [42].

## 5. Conclusions

In conclusion, an elevated antioxidant capability and a lower concentration of inflammatory molecules is required to obtain good fertilization rates, top-quality embryos, and embryo implantation.

Within the limits of our study, these results further underlined the need for standardized protocols of IVF and for quantifying the predictive FF metabolites. Determination of the metabolites in FF may be useful for understanding oocyte fertilization and embryo quality, and predicting IVF outcomes. These investigations may be helpful to develop effective pharmacological treatments. According to our results, oxidative stress and the consequent modulation of the antioxidant potential of FF may represent a key point to manage fertilization and the quality of oocytes. According to the literature, both antioxidant supplementation and management of the embryo culture conditions may help improve the final quality of embryos obtained during IVF procedures [43,44]. In this sense, Luddi et al. have demonstrated that the treatment based on micronutrient supplementation (starting 3 months before the IVF cycles) can protect the follicular microenvironment from oxidative stress, thus increasing the number of good-quality oocytes recovered during retrieval and finally improving the IVF outcome [45].

## Figures and Tables

**Table 1:** Hormonal and clinical parameters measured in the women undergoing IVF treatment. Differences between unfertilized (NF) and fertilized (F) oocytes, and between poor-quality (NTQ) and top-quality embryos (TQ) are shown for the analyzed parameters: age, body mass index (BMI), follicle-stimulating hormone levels (FSH), luteinizing hormone levels (LH), serum levels of estradiol (E2), anti-Müllerian hormone levels (AMH), antral follicle count (AFC), male age, total units of gonadotropins, days of stimulation, progesterone and estradiol levels, and number of follicles > 16 mm on the trigger day, number of oocytes collected, number of mature oocytes MII, number of fertilized oocytes, number of top-quality embryos, and positive β-HCG values. All the data are expressed as means ± SD. Statistical analysis was performed by unpaired *t*-tests, considering the following pairs: F vs. NF and TQ vs. NTQ. ^#^ *p* < 0.05 vs. NTQ.

Parameters	NF (N = 15)	F (N = 92)	NTQ (N = 37)	TQ (N = 55)
Age (years)	34.80 ± 1.74	35.64 ± 1.76	35.77 ± 1.85	35.80 ± 1.67
BMI (kg/m^2^)	24.09 ± 4.29	23.01 ± 4.60	22.86 ± 4.88	23.09 ± 4.54
FSH (IU/mL)	8.40 ± 2.14	8.42 ± 2.86	7.91 ± 2.23	8.75 ± 3.24
LH (mIU/mL)	6.19 ± 2.49	5.80 ± 2.56	5.75 ± 2.54	5.88 ± 2.64
Estradiol (pg/mL)	55.36 ± 28.95	43.85 ± 20.33	44.15 ± 20.55	44.60 ± 20.55
AMH (ng/mL)	2.88 ± 1.81	2.56 ± 1.77	2.80 ± 1.85	2.44 ± 1.73
AFC	12.85 ± 5.55	10.98 ± 4.26	11.78 ± 4.03	10.43 ± 4.32
Male age (years)	38.27 ± 3.26	38.35 ± 3.71	38.17 ± 3.91	38.68 ± 3.57
Gonadotropins (UI)	2896.67 ± 910.10	3220.99 ± 1286.33	3322.94 ± 1401.32	3182.46 ± 1247.70
Days of stimulation	9.33 ± 1.72	9.58 ± 2.28	9.71 ± 2.35	9.52 ± 2.25
Progesterone on the trigger day (ng/mL)	1.19 ± 0.61	1.72 ± 0.91	1.40 ± 0.75	1.22 ± 0.61
Estradiol on the trigger day (pg/mL)	1990.27 ± 1557.12	2291.31 ± 1834.63	2662.03 ± 1975.31	2075.94 ± 1752.01
Follicles > 16 mm on the trigger day	5.27 ± 2.63	4.67 ± 2.19	5.15 ± 2.22	4.31 ± 2.04
Oocytes collected	4.40 ± 1.88	4.86 ± 2.37	5.40 ± 2.30	4.52 ± 2.42
Mature oocytes MII	4.07 ± 1.58	4.10 ± 1.89	4.54 ± 1.77	3.81 ± 1.94
Fertilized oocytes	-	3.64 ± 1.99	4.23 ± 1.78	3.26 ± 2.08 ^#^
Top-quality embryos	-	-	-	1.44 ± 1.00
Positive β-HCG	-	26.09	13.51	34.55

**Table 2:** Biochemical parameters measured in the follicular fluid (FF) collected from unfertilized (NF) and fertilized (F) oocytes, distinguished as poor-quality (NTQ) or top-quality (TQ) embryos. IL-6, IL-8, IL-10, and IL-12 are expressed as pg/5 µg of total proteins in the FF. HIF-1α and NF-kB are expressed as pg/5 µg total proteins in the FF, while TGF-β is expressed as ng/5 µg of total proteins in FF. TOSC values versus hydroxyl or peroxyl radicals are expressed as TOSC units. All the data are expressed as means ± SD. Statistical analysis was performed by unpaired *t*-tests between NF and F, and between TQ and NTQ. * *p* < 0.05, ** *p* < 0.01, *** *p* < 0.001 vs. NF; ^#^ *p* < 0.05, ^##^ *p* < 0.01, ^###^ *p* < 0.001 vs. NTQ.

Parameter	NF (Mean ± SD)	F (Mean ± SD)	NTQ (Mean ± SD)	TQ (Mean ± SD)
IL-6 (pg/5 µg total proteins)	1851.32 ± 491.23	1171.53 ± 665.56 ***	1380.26 ± 606.31	1010.32 ± 646.47 ^##^
IL-8 (pg/5 µg total proteins)	82.93 ± 20.82	63.37 ± 32.08 *	79.12 ± 31.47	53.68 ± 28.56 ^###^
IL-10 (pg/5 µg total proteins)	10.58 ± 8.00	48.97 ± 40.77 ***	25.21 ± 13.54	64.97 ± 45.48 ^###^
IL-12 (pg/5 µg total proteins)	44.60 ± 31.20	20.48 ± 17.82 ***	29.46 ± 24.44	15.22 ± 8.67 ^###^
HIF-1α (pg/5 µg total proteins)	190.65 ± 153.88	108.39 ± 80.79 **	164.59 ± 97.07	74.06 ± 38.52 ^###^
TGF-β (ng/5 µg total proteins)	56.10 ± 14.56	39.40 ± 9.96 ***	44.51 ± 10.28	36.02 ± 8.39 ^###^
NF-kB (pg/5 µg total proteins)	447.65 ± 293.55	303.31 ± 266.35	402.65 ± 294.98	237.02 ± 229.11 ^##^
TOSC vs. peroxyl radicals	8.61 ± 1.97	14.03 ± 4.70 ***	12.35 ± 3.16	14.94 ± 5.24 ^#^
TOSC vs. hydroxyl radicals	5.96 ± 2.51	8.08 ± 3.65 *	6.69 ± 2.58	9.03 ± 3.97 ^##^

**Table 3:** Accuracies of each FF biomarker investigated in discriminating fertilized oocytes from unfertilized ones (**a**), and top-quality embryos from poor-quality ones (**b**). The area under the receiver operating characteristic curve (AUC) and its confidence interval (CI) are reported.

(**a**)			
**Group comparisons**	**Predictors**	**AUC**	**95% CI**
Fertilized vs. unfertilized oocytes	IL-6	0.2433	0.1229–0.3567
	IL-8	0.2600	0.1232–0.3871
	IL-10	0.9100	0.7922–0.9624
	IL-12	0.2854	0.1106–0.4431
	HIF-1α	0.3462	0.1646–0.5051
	TGF-β	0.1646	0.0233–0.2994
	TOSC vs. peroxyl radicals	0.8533	0.7593–0.9125
	TOSC vs. hydroxyl radicals	0.7146	0.5475–0.8269
(**b**)			
**Group comparisons**	**Predictors**	**AUC**	**95% CI**
Top-quality vs. poor-quality embryos	IL-6	0.3007	0.1822–0.4106
	IL-8	0.2263	0.1221–0.3256
	IL-10	0.8241	0.7026–0.8989
	IL-12	0.3597	0.2245–0.4813
	HIF-1α	0.1831	0.0798–0.2826
	TGF-β	0.2467	0.1271–0.3593
	TOSC vs. peroxyl radicals	0.6877	0.5469–0.7907
	TOSC vs. hydroxyl radicals	0.7332	0.5972–0.8282

## Data Availability

Data is contained within the article.
